# Supplementary material for: Resolving the full spectrum of human genome variation using Linked-Reads
Source: Genome Res. 2019 Apr;29(4):635–45. doi: 10.1101/gr.234443.118 (PMC6442396; doi:10.1101/gr.234443.118)
Supplement: Supplemental Material [file supp_gr.234443.118_Supplemental_longranger-2.2.zip › longranger-2.2.tar.bz2!/lr2.2-public/lib/stan/cmdstan/stan/doc/api/html/fwd_2functions_2atan2_8hpp_source.html]

Stan: src/stan/agrad/fwd/functions/atan2.hpp Source File


|  |  |
| --- | --- |
|  | Stan  2.5.0  probability, sampling & optimization |


- Main Page
- Related Pages
- Namespaces
- Classes
- Files

- File List
- File Members

All Classes Namespaces Files Functions Variables Typedefs Enumerations Enumerator Friends Macros Pages

- src
- stan
- agrad
- fwd
- functions

atan2.hpp

Go to the documentation of this file.

1 #ifndef STAN\_\_AGRAD\_\_FWD\_\_FUNCTIONS\_\_ATAN2\_HPP

2 #define STAN\_\_AGRAD\_\_FWD\_\_FUNCTIONS\_\_ATAN2\_HPP

3

4 #include <stan/agrad/fwd/fvar.hpp>

5 #include <stan/meta/traits.hpp>

6 #include <stan/math/functions/square.hpp>

7 #include <math.h>

8

9 namespace stan {

10

11  namespace agrad {

12

13  template <typename T>

14  inline

15  fvar<T>

16  atan2(const fvar<T>& x1, const fvar<T>& x2) {

17  using ::atan2;

18  using stan::math::square;

19  return fvar<T>(atan2(x1.val\_, x2.val\_),

20  (x1.d\_ \* x2.val\_ - x1.val\_ \* x2.d\_) /

21  (square(x2.val\_) + square(x1.val\_)));

22  }

23

24  template <typename T>

25  inline

26  fvar<T>

27  atan2(const double x1, const fvar<T>& x2) {

28  using ::atan2;

29  using stan::math::square;

30  return fvar<T>(atan2(x1, x2.val\_),

31  (-x1 \* x2.d\_) / (square(x1) + square(x2.val\_)));

32  }

33

34  template <typename T>

35  inline

36  fvar<T>

37  atan2(const fvar<T>& x1, const double x2) {

38  using ::atan2;

39  using stan::math::square;

40  return fvar<T>(atan2(x1.val\_, x2),

41  (x1.d\_ \* x2) / (square(x2) + square(x1.val\_)));

42  }

43  }

44 }

45 #endif

stan::math::square

T square(const T x)

Return the square of the specified argument.

**Definition:** square.hpp:22

stan::agrad::fvar

**Definition:** fvar.hpp:13

square.hpp

stan::agrad::square

fvar< T > square(const fvar< T > &x)

**Definition:** square.hpp:15

traits.hpp

fvar.hpp

stan::agrad::atan2

var atan2(const double a, const var &b)

Return the principal value of the arc tangent, in radians, of the first scalar divided by the second ...

**Definition:** atan2.hpp:121

stan::agrad::atan2

fvar< T > atan2(const fvar< T > &x1, const fvar< T > &x2)

**Definition:** atan2.hpp:16

stan::agrad::fvar::d\_

T d\_

**Definition:** fvar.hpp:16

stan::agrad::fvar::val\_

T val\_

**Definition:** fvar.hpp:15


---

[ Stan Home Page ]

*© 2011–2014,
Stan Development Team.*
